# Draft Genome Sequence of *Shewanella decolorationis* S12, a Dye-Degrading Bacterium Isolated from a Wastewater Treatment Plant

**DOI:** 10.1128/genomeA.00993-13

**Published:** 2013-12-05

**Authors:** Meiying Xu, Yun Fang, Jun Liu, Xingjuan Chen, Guoping Sun, Jun Guo, Zhengshuang Hua, Qichao Tu, Liyou Wu, Jizhong Zhou, Xueduan Liu

**Affiliations:** Guangdong Institute of Microbiology, Guangzhou, Guangdong, Chinaa; School of Minerals Processing and Bioengineering, Central South University, Changsha, Hunan, Chinab; Key Laboratory of Biometallurgy of Ministry of Education, Changsha, Hunan, Chinac; State Key Laboratory Cultivation Base, Province & Ministry Co-constructed South China Applied Microbiology Laboratory, Guangzhou, Guangdong, Chinad; State Key Laboratory of Biocontrol and Guangdong Provincial Key Laboratory of Plant Resource, School of Life Sciences, Sun Yat-Sen University, Guangzhou, Chinae; Institute for Environmental Genomics, and Department of Botany and Microbiology, Stephenson Research & Technology Center, University of Oklahoma, Norman, Oklahoma, USAf

## Abstract

*Shewanella decolorationis* is a valuable microorganism for degrading diverse synthetic textile dyes. Here, we present an annotated draft genome sequence of *S. decolorationis* S12, which contains 4,219 protein-coding genes and 86 structural RNAs. This information regarding the genetic basis of this bacterium can greatly advance our understanding of the physiology of this species.

## GENOME ANNOUNCEMENT

The genus *Shewanella* is famous for bioremediation of environments contaminated with toxic organic and inorganic chemicals and has been found in water, sediment, and surfaces of shale and sandstone (1). Recent studies have demonstrated that *Shewanella decolorationis* is a powerful species to degrade diverse manufactured textile dyes, including azo and anthraquinone dye, which are carcinogenic to humans (2–4). *S. decolorationis* S12 was isolated in 2002 from activated sludge of a textile-printing wastewater treatment plant in Guangzhou, China (3). Whole-genome sequences of *S. decolorationis* have not been available, which is a limitation to the genetic study of this useful species.

Genomic DNA extracted from *S. decolorationis* S12 was sequenced on an Illumina Miseq sequencer according to Illumina’s recommendations. A total of 16,692,489 paired-end 250-base reads were generated. The draft genome was assembled using Velvet (5) and Newbler, containing 77 contigs of >250 bp each after manual curation, with a total of 4,843,449 bases, an *N*_50_ of 155.5 kbp, and a G+C content of 47.1%. The draft genome sequence of strain S12 was annotated using Blast2GO (6) and the NCBI Prokaryotic Genome Automatic Annotation Pipeline (PGAAP) (7, 8).

The draft genome sequence contains 4,305 genes, including 4,219 predicted coding sequences (CDS), 9 rRNA genes, and 77 tRNA genes. A total of 3,184 CDS were classified using the Cluster of Orthologous Groups (COG) database (9). The most abundant groups of proteins were those that are involved in signal transduction (COG category T [9.5%]), amino acid metabolism and transport (COG category E [9.0%]), transcription (COG category K [8.5%]), and energy production and conversion (COG category C [7.8%]). The genome sequence of *S. decolorationis* S12 serves as a basis for further investigation of the molecular basis of its potential in dye degradation.

Currently, the genus *Shewanella* contains 45 type species, and 28 strains from 15 type species have been sequenced for the whole genomes. Unlike these species from natural environments, *S. decolorationis* S12 was isolated from a human-altered and highly contaminated system, and thus its genetic evolution and cell responses might be different. Comparative genomics analysis showed that *S. decolorationis* S12 was closest to *Shewanella* sp. strain ANA-3 (89% coding sequence similarity), followed by *Shewanella* sp. strain MR-7 (88%) and *Shewanella* sp. strain MR-4 (88%). Further analysis will provide a better understanding of the *S. decolorationis* genome and shed more light on genomic evolution of the *Shewanella* genus.

### Nucleotide sequence accession numbers.

This whole-genome shotgun project has been deposited at DDBJ/EMBL/GenBank under accession number AXZL00000000. The version described in this paper is the first version, AXZL01000000.
